# Case of Tubercular Meningitis, Occurring at St. Joseph’s Hospital

**Published:** 1850-03

**Authors:** 


### MEDICAL EXAMINER,

AND

### RECORD OF MEDICAL SCIENCE.

NEW SERIES.—NO. LXIII.—MARCH, 1850.

---

### ORIGINAL COMMUNICATIONS.

---

### *Case of Tubercular Meningitis, occurring at St. Joseph's Hospital.*

Service of Dr. Stillé.

Catharine S., aged about 12 years, entered the hospital Oct., 1849. Catharine arrived in this country from Ireland, in Dec., 1848, with her parents and several younger children. Very soon afterwards both father and mother died from the effects, it is supposed, of a long voyage and insufficient food. Catharine was their only nurse, and watched them day and night with a devotion and intelligence far beyond her years. She was completely exhausted by her labors, and at her mother's funeral, which she would not be prevented from attending, could only succeed in following the coffin by interrupted and persevering efforts. On returning home she fainted repeatedly. She was then taken into the employment of a benevolent lady, with whom she continued to live. She remained feeble, however, and complained of weakness in her limbs, and of a sense of tightness around her waist. Within the last month or two, her eyes gave her pain whenever she applied them to needlework, although no signs of inflammation were visible; she suffered almost constantly from headache, and at times betrayed a degree of perverseness foreign to her natural disposition. About a week before entering the hospital she was attacked by spasmodic movements, with some wandering of the mind, and stupor, which were regarded by her physician as symptoms of hysteria, and treated accordingly. After her admission she lay with closed eyes, her brow somewhat corrugated, and her cheeks flushed. With much difficulty she could be roused, so as to answer questions by "yes" or "no," but immediately relapsed into stupor. The natural temperature of the skin was preserved, and the pulse was frequent and feeble. She refused nutritious food, but ate greedily portions of an apple or orange placed in her mouth. There was a partial paralysis of the whole of the left side of the body. The coma was not uniformly of the same degree. Occasionally it was interrupted by sharp and sudden cries, with grinding of the teeth; at night, by active delirium, and once or twice, in the morning, by a state of comparative consciousness, in which she spoke some sentences connectedly. The latter symptom followed the application of a large blister to the nuchæ. Calomel, and other bulky purgatives, administered by the mouth, produced vomiting; croton oil, which was freely prescribed, was retained, but without evacuating the bowels. The blistered surface was dressed with mercurial ointment.

*Oct. 3d.*—Face sunken; left side partially paralyzed; the right eye slightly sensible to light, the left not at all. Pulse so feeble that it cannot be counted; pulsations of the heart 160 in a minute, and irregular; no intelligence; the eyes injected and filmy; can scarcely swallow; automatic movements of the hands, but chiefly of the right hand; passes her water in the bed; no stool.

This state continued until early on Oct. 4th, when she died gradually, and by exhaustion.

*Autopsy,* 12 hours after death. Rigidity of the limbs and emaciation moderate; skin sallow, but not jaundiced; no ecchymoses in the depending parts.

*Head.*—Scalp dry, with little fat; no blood followed its incision. On removing the calvarium, a few drops only of dark blood flowed. Vessels of dura mater distended. Surface of arachnoid dry, sticky, and looking as if varnished. Its opposite surfaces were adherent at four or five points on either side of the longitudinal sinus, where there were somewhat firm and opaque fibrinous deposits. The vessels of the pia mater were uniformly injected; the smaller of a dusky reddish hue, and the larger veins along the anfractuosities swollen and of a leaden color. About two ounces of limpid serum were at the base of the brain and in the opening of the spinal canal. The vessels of the base were injected like those of the upper surface of the brain.

A semitransparent, thick and dense layer of fibrine covered the chiasm of the optic nerves, extending along the fissure of Silvius, and the angle between the cerebellum and the pons varolii and medulla oblongata, on *both* sides. These adjacent parts were thus firmly connected, and the middle could not be separated from the anterior lobe by traction, without tearing the substance of the former. In this fibrinous deposit were numerous granular bodies, scarcely exceeding half a line in diameter, more opaque than the substance around them, but neither hard nor readily isolated from this substance. They were most abundant in the fissure of Silvius, and along the edge of the medulla oblongata and pons varolii, but also existed in the choroid plexus and the velum interpositum.

The lateral ventricles were distended with serum, and the cerebral substance between them was in some degree softened. The *internal face of the right optic thalamus was distinctly softened* in the space of half an inch square. The superior part of the hemispheres retained their normal consistence, but the base was less firm than natural. The two constituents of the cerebral mass contrasted strongly in color; the medullary portion was very white, but presented upon section innumerable dark bloody points.

*Thorax.*—The cutis was remarkably thin, with a very slight layer of subcutaneous fat. All of the superficial tissues, including the muscles, were remarkably dry. The pleural cavities and the pericardium contained no fluid. The lungs were of a perfectly natural aspect upon their anterior face, and crepitated freely when handled. The posterior surface of the lower lobe of the left lung was mottled with black spots upon a ground the color of logwood. The latter color was due to general hypostatic congestion, and the former to local circumscribed effusions of blood, about the size of an orange seed. The corresponding portion of the right lung was more thoroughly congested, its natural toughness was somewhat impaired, and a fragment of it partially sunk in water.

From several points of a section of this part pus flowed upon pressure. The bronchiæ were dyed of a dark red color: the bronchial glands were unchanged. No tubercles were detected in either lung.

*Heart.*—Its right cavities were distended with black blood, its left empty. In all other respects the organ was normal.

*Abdomen.*—The general color of the intestinal tube was a dusky red. The peritoneum dry and dull. The liver extended completely to the left hypochondrium, and its right lobe was unusually thick and had a pyramidal shape. Its lower surface presented a fissure running nearly parallel with the edge, about an inch deep at one point, and looking precisely like a cut made with a scalpel. The peritoneum over it was quite natural. The general color of the liver was very dark, and its surface mottled with lighter spots in the general dark ground: a section presented a similar aspect. The whole organ was gorged with black blood, and had less firmness than natural. The gall bladder was filled with very dark bile about the consistence of molasses.

The mesenteric glands were undeveloped, and the glands of Peyer in the ileum could only be distinguished by a reflected light. The lining membrane of this bowel was of a dusky red color. The caput coli was found in the lower pelvis, whence the ascending colon rose in front of and then to the *left* of the spine, till it reached the pancreas, where it made an abrupt turn upon itself and descended, in contact with the ascending portion, to the left iliac region, whence it again rose to the neighborhood of the spleen, and once more pursued the downward and usual course. The part between the caput coli and the pancreas was distended with gas, and hardened fæces could be felt within it. The following divisions were contracted, but contained a portion of fæces.

The uterus was scarcely thicker than a dollar, and its body about an inch in length by ¾ of an inch broad.

The other viscera were not examined.

The points of interest in this case are the coincidence of paralysis of the left side and acute softening of the right optic thalamus; the size and singular shape of the liver; and the malposition of the colon, which may plausibly be attributed to the morbid development of the liver. It is probable that the stricture around the waist which the patient complained of may also be attributed to the condition of the liver.

